# Shortened Palliative Radiotherapy Results in a Lower Rate of Treatment During the Last Month of Life

**DOI:** 10.7759/cureus.21617

**Published:** 2022-01-25

**Authors:** Carsten Nieder, Ellinor C Haukland, Bård Mannsåker

**Affiliations:** 1 Oncology, Nordland Hospital Trust, Bodø, NOR; 2 Translational Cancer Research Group, The University of Tromsø (UiT) - The Arctic University of Norway, Tromsø, NOR; 3 Department of Quality and Health Technology, SHARE-Center for Resilience in Healthcare, University of Stavanger, Stavanger, NOR

**Keywords:** metastatic cancer, fractionation, end of life care, treatment completion, quality of care, overtreatment, palliative radiation therapy

## Abstract

Introduction

Palliative radiotherapy (PRT) during the last month of life (PRT30) should be avoided because relevant clinical benefits are unlikely to occur. While traditional short-course fractionation regimens are suitable for most patients, a minority may derive gains from higher doses of PRT. Compared to older regimens such as 13 fractions of 3 Gy, more hypofractionated, non-ablative concepts with reduced overall treatment time are not well studied.

Methods

Retrospective analysis (2017-2020) of 107 patients treated to metastatic lesions (one or two target volumes per patient) with traditional >2 weeks regimens or newer ≤2 weeks regimens, e.g. seven fractions of 5 Gy or five fractions of 6 Gy.

Results

Failure to complete radiotherapy was registered in 8% of patients (traditional fractionation) and 1%, respectively (p=0.12). Moderate rates of PRT30 were observed (11% and 6%, respectively, p=0.44). PRT30 was more likely in patients irradiated for brain or lymph node metastases. Utilization of newer ≤2 weeks regimens was highest in 2020, presumably as a result of the coronavirus disease 2019 (COVID-19) pandemic.

Conclusion

The implementation of newer fractionation regimens for selected patients has resulted in acceptable rates of non-completion and PRT30. Optimal selection criteria remain to be determined. Established, guideline-endorsed short-course regimens such as five fractions of 4 Gy and 8-Gy single fractions continue to represent important PRT approaches.

## Introduction

Standard palliative radiotherapy (PRT) regimens with a long track record include a single fraction of 8 Gy, five fractions of 4 Gy, and 10 fractions of 3 Gy [1-4]. If the primary aim of treatment is symptom palliation, priority is given to convenient, short-course regimens, which avoid toxicity [5]. In selected patients, 13 fractions of 3 Gy and comparable regimens have been preferred if increased tumor cell killing is desirable, which potentially increases time to in-field failure [6]. With prolonged overall treatment time, the risks of disease progression outside the irradiated area or clinical deterioration increases due to various causes, resulting in a higher likelihood of premature termination of the planned treatment course. Despite advanced techniques such as simultaneous integrated boost (SIB) intensity-modulated radiotherapy (IMRT) or volumetric modulated arc treatment (VMAT), overall treatment time may still be longer than two weeks [7,8]. Hypofractionation with doses per fraction in the range of 5-8 Gy may decrease overall treatment time to two weeks or less for patients who are likely to experience the potential benefit of an equivalent dose (EQD2) that is higher than that of 10 fractions of 3 Gy. At the extreme end of the specter, single fraction ablative radiotherapy e.g. stereotactic ablative body radiotherapy (SABR) or stereotactic radiosurgery (SRS) of brain metastases represent both convenient (minimal overall treatment time) and efficacious (high EQD2) regimens [9,10]. Due to target volume size and proximity to nearby organs at risk, which may preclude safe single fraction SABR/SRS, fractionated treatment with a relatively high total dose continues to play an important role in daily practice.

The administration of palliative radiotherapy very close to the end of life, e.g. the last 30 days (PRT30), has been under scrutiny in recent years because futile treatment afflicts both patients, caregivers, and healthcare providers [11-13]. Several studies have evaluated utilization rates in different settings. In the authors’ institution, which serves a small and scattered population of fewer than 200,000 inhabitants in rural North-Norway, overtreatment and 30-day mortality have long been a topic of research. Initially, the time period 2007-2009 was evaluated, i.e. the starting phase after the opening of our facility [14], the rate of PRT30 was 9%. In March 2020, the global COVID-19 pandemic also arrived in Norway. Based on national recommendations distributed to all oncology departments, we encouraged the utilization of altered fractionation regimens with a focus on short overall treatment time, as well as a thorough assessment of the potential benefits of palliative radiotherapy, in line with suggestions developed by international groups [15,16]. Our group increased the prescription of SABR-like hypofractionated regimens with non-ablative doses in patients previously managed with 13 or more fractions of PRT. We also adopted the single fraction 12-or 16-Gy concept for bone metastases not suitable for a single fraction of 8 Gy published by Nguyen et al. [17]. As already reported, in the first half of 2020 still 9% of our patients received PRT during the final 30 days of life [18]. The aim of the present analysis was to compare the rate of PRT30 with the traditional >2 weeks regimens to that of the newer ≤2 weeks regimens, both of whom prescribed to selected patients with an estimated survival justifying a two-goal concept (symptom palliation combined with in-field control). 

## Materials and methods

This retrospective single-institution study employed the following inclusion criteria: adult patients (actual age ranging from 44 to 93 years), metastatic solid tumor, irradiation of metastatic sites in routine clinical practice outside of prospective trials, and consecutively treated in the four-year time period (2017-2020). Exclusion criteria were as follows: irradiation of symptomatic primary tumors, e.g. lung cancer, bladder cancer, primary brain tumors; standard palliative regimen such as 10 fractions of 3 Gy; ablative radiotherapy of intact metastases with high EQD2, e.g. SRS; post-operative radiotherapy, e.g. of surgical cavities after resection of brain metastases. All treatments had EQD2 <90 Gy (alpha/beta-value 10 Gy, calculated according to the linear-quadratic model [19]). Descriptive statistics and two-tailed Fisher’s exact probability tests were employed for statistical analyses in IBM SPSS statistics version 27.0 (IBM Corp., Armonk, NY). Survival data were obtained in September 2021 by use of the hospital’s electronic patient records. The latter were also utilized to extract baseline characteristics. 

## Results

**Figure 1:**
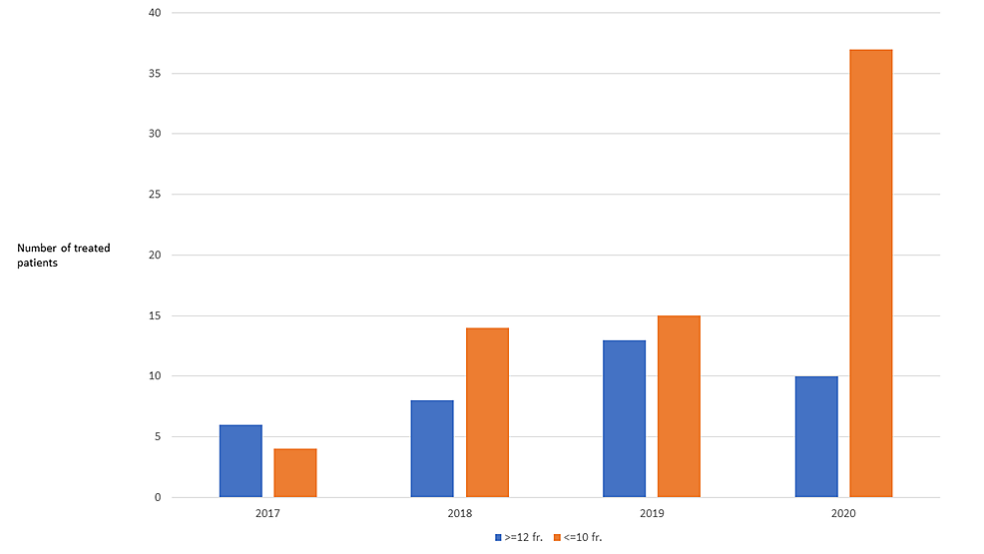
Utilization of different fractionation regimens between 2017 and 2020

The total number of patients treated with traditional fractionation regimens such as 13 fractions of 3 Gy was 37, while 70 patients received newer regimens. As illustrated in Figure 1, the utilization of any such regimen has increased steadily after 2017. 

The proportion of patients treated with newer regimens in 2020 was larger than ever before. The median age was 69 years (range 44-85) in the traditional fractionation group (74 years, range 45-93, in the other group). While most patients in the traditional fractionation group received 13 fractions (68%) and 3 Gy per fraction (76%), a wide variety of regimens was prescribed in the other group (Table 1). 

**Table 1:** Comparison between patients treated with traditional palliative radiotherapy >10 fractions (n=37) and patients treated with newer palliative radiotherapy regimens ≤10 fractions (n=70): baseline parameters SCLC: small cell lung cancer, NSCLC: non-small cell lung cancer, * fraction size 2.5 or 3 Gy

Parameter	>10 fractions (number, percent)	≤10 fractions (number, percent)
Gender		
Female sex	18, 49%	25, 36%
Male sex	19, 51%	45, 64%
Primary disease site		
Breast cancer	2, 5%	0
Prostate cancer	5, 14%	10, 14%
SCLC	2, 5%	5, 7%
NSCLC	7, 19%	22, 31%
Kidney cancer	3, 8%	8, 11%
Bladder cancer	3, 8%	3, 4%
Gastrointestinal cancer	11, 30%	8, 11%
Sarcoma	2, 5%	3, 4%
Gynecological cancer	2, 5%	0
Malignant melanoma	0	9, 13%
Others	0	2, 3%
Site of treated metastasis		
Bone metastasis target	12, 32%	25, 36%
Lymph node metastasis target	16, 16%	6, 9%
Brain metastasis target	3, 8%	18, 26%
Skin or soft tissue target	2, 5%	9, 13%
Lung metastasis target	3, 8%	6, 9%
Adrenal metastasis target	1, 3%	6, 9%
Number of fractions		
12 fractions	4, 11%	-
13 fractions	25, 68%	-
14 fractions*	4, 11%	-
≥15 fractions	4, 11%	-
Single fraction	-	8, 11%
3-4 fractions	-	7, 10%
5 fractions	-	16, 23%
6 fractions	-	4, 6%
7 fractions	-	21, 30%
8 fractions	-	4, 6%
9-10 fractions	-	10, 14%
Median age (years), range	69, 44-85	74, 45-93
Median Karnofsky performance status, range	80, 60-100	80, 60-100

Eight patients (11%) received single fractions of 12 or 16 Gy to bone metastases. Twenty-seven patients (39%) received 5-Gy fractions (often 5 Gy x7) and 17 patients (24%) received 6-Gy fractions (often 6 Gy x5). The majority of targets consisted of bone, brain, and lymphatic metastases. As also shown in Table 1, patients with malignant melanoma or kidney cancer were commonly treated with one of the newer, more hypofractionated regimens.

Failure to complete radiotherapy was registered in 3/37 patients (traditional fractionation, 8%) and 1/70 patients (newer regimens, 1%), respectively (p=0.12). The figures for treatment in the last month of life were 4/37 (11%) and 4/70 (6%), respectively, p=0.44 (Table 2). 

**Table 2:** Comparison between patients treated with traditional palliative radiotherapy >10 fractions (n=37) and patients treated with newer palliative radiotherapy regimens ≤10 fractions (n=70): outcomes PRT30: palliative radiotherapy in the last month of life (all patients had a sufficiently long follow-up to assess this endpoint) *patients with ongoing follow-up <1 year were excluded for this endpoint because they were at risk of shorter survival

Parameter	>10 fractions (number)	>10 fractions (percent)	≤10 fractions (number)	≤10 fractions (percent)
PRT30	4	11	4	6
PRT last 2 weeks of life	2	5	1	1
Incomplete PRT	3	8	1	1
Alive 1 year after PRT*	16/36	44	19/64	30

When looking at combined data from all 107 patients, the median overall survival was 10.8 months (minimum 0.5, maximum 47 in a patient who is currently alive). It is interesting to note that male patients were more likely to receive radiotherapy in the last month of life (seven of eight patients were males). Four of eight had brain metastases (three had lymphatic and one bone metastases). Regarding primary tumor type and age, no particular trends emerged (three non-small cell lung cancer, two prostate cancer, one sarcoma, one colon cancer, one small cell lung cancer; age range 46-93 years).

## Discussion

Patients receiving palliative radiotherapy for metastatic cancers are at risk of unnecessary overtreatment in the last month of life. For example, 24% of patients were treated within 30 days to death in one study [11]. According to a systematic review, the overall palliative radiotherapy utilization rates during the last month of life were lower (in the range of 5-10%) among patients who died of cancer [12]. The most commonly used regimen was 10 fractions of 3 Gy (36-90%). Single fraction utilization ranged from 0 to 59%. Given that our institution regularly has evaluated this quality of care indicator and found largely stable rates despite efforts to improve the prediction of short survival [14,18], we gradually implemented newer altered-fractionation regimens to shorten the overall treatment time. While the vast majority of our patients receive traditional short-course regimens such as 8 Gy x1 or 4 Gy x5, a selected proportion of patients with good performance status (mostly 0-2) who are not candidates for ablative SABR/SRS approaches represent a group that is not always easy to manage. On one hand, the referring oncologists believe that these patients likely will survive long enough to benefit from a certain in-field local control, on the other hand, dynamic changes in out-of-field tumor growth, performance status, and prognostically relevant blood test results may occur at any time, thus turning local treatment into a futile effort.

In the immediate past, in line with other institutions [2], many patients selected for higher than traditional palliative doses were prescribed 12-15 fractions, including the 13 fractions of 3 Gy regimen in three-dimensional conformal technique, as already shown in Table 1. More recently, 3-7 fraction regimens delivered by IMRT or VMAT approaches have been utilized in many patients. This transition began before the COVID-19 pandemic. However, our recent pre-versus post-COVID-19 analysis showed reduced utilization of palliative radiotherapy with 10 or more fractions (p=0.008) during the pandemic [18]. The markedly higher number of patients treated with non-ablative, high-EQD2 regimens in 2020, which is evident from Figure 1, likely is a result of the COVID-19 pandemic, which has led to deferred systemic treatment in patients with upfront oligometastases or oligoprogression during a drug holiday. 

The main goal of the present analysis was to compare the rate of PRT30 with the traditional >2 weeks regimens to that of the ≤2 weeks regimens. It was reassuring to see a low rate of 6% with newer altered-fractionation concepts. Despite the lack of statistical significance, the results displayed in Table 2, e.g. high rate of treatment completion, support the continued implementation of short-course regimens with higher EQD2 than the traditional 5-10 fraction regimens, provided prospectively generated evidence confirms benefits in terms of patient-reported outcomes, symptom control and in-field control. Of course, cost-effectiveness, which depends on the healthcare and reimbursement environment, should be assessed too. Savings resulting from the reduced number of fractions might be outweighed by the cost of IMRT or VMAT replacing simpler three-dimensional conformal techniques. The fact that our one-year survival rates were <50% illustrates the continued challenge in patient selection and survival prediction. In patients with limited survival, a three-week course of radiotherapy (13-15 fractions) is not warranted. According to Wu et al., palliative radiotherapy within 30 days of death was associated with older age, shorter intervals since diagnosis, liver metastasis, lower performance status, lower body mass index, and inpatient status at consult [11].

If a study includes other potentially predictive parameters results may change, as indicated by a previous analysis of own data [20]. In that study, patients with metastatic cancer had higher rates of PRT30 in case of lower performance status, liver metastases, brain metastases, bone metastases, pleural metastases (or effusion), leukocytosis, elevated C-reactive protein, more than one cancer diagnosis, progressive disease outside the intended radiotherapy region, pain management with opioids, and ongoing steroid medication.

There is still debate about the complete avoidance of palliative radiotherapy in the last month of life. Recently, Christ et al. reported that radiotherapy achieved high completion and success rates until one week before death, and suggested that treatment within one week of death should be restricted to carefully selected patients or avoided altogether [21]. In a different study, treatment was discontinued in 41% of the patients irradiated during the last two weeks of life, and worsening of the general condition was the prevailing reason for discontinuation (70%) [22]. Our own policy is quite restrictive, as we try to avoid treatment in the whole last month, acknowledging that a low PRT30 rate may be more appropriate than aiming at 0%. We recommend assessing prognostic models such as the TEACHH score [23] and the LabBM score, which is based on inexpensive routine blood tests (hemoglobin, platelets, albumin, C-reactive protein, lactate dehydrogenase) [24]. Patients assessed with the LabBM score at our hospital had a median survival of 1.1 months only, if all five blood tests were abnormal. At present, we do not use any of the prognostic models as a singular decision criterion when accepting patients for palliative radiotherapy. However, we believe they are helpful in avoiding crude mismatch between treatment intensity and remaining life span.

The present study has both strengths, such as the routine clinical practice setting, and limitations, such as the low statistical power resulting from small group sizes, and limited follow-up if treatment was administered in 2020. Larger national or regional studies are warranted to analyze the pros and cons of non-ablative innovative hypofractionation concepts. If safe administration is possible, a single fraction of 16 Gy is more convenient than five fractions of 6 Gy. From the efficacy point-of-view, ablative total doses (EQD2 >100 Gy) may be considered oncologically safer than lower doses that often correspond to an EQD2 of 50-75 Gy. However, continued local control after 2-3 years is relevant only if the progression of non-irradiated sites of disease can be inhibited. Furthermore, one must be aware of the risk of toxicity that comes with dose escalation. In observation of the primum non nocere principle, prescription of traditional short-course regimens should always be considered when planning palliative radiotherapy. 

## Conclusions

The implementation of newer fractionation regimens for selected patients has resulted in acceptable rates of non-completion and PRT30. Optimal selection criteria remain to be determined. Established, guideline-endorsed short-course regimens such as five fractions of 4 Gy and 8-Gy single fractions continue to represent important PRT approaches.
